# Tracking microevolution events among ST11 carbapenemase-producing hypervirulent *Klebsiella pneumoniae* outbreak strains

**DOI:** 10.1038/s41426-018-0146-6

**Published:** 2018-08-12

**Authors:** Ning Dong, Xuemei Yang, Rong Zhang, Edward Wai-Chi Chan, Sheng Chen

**Affiliations:** 1 Shenzhen Key Lab for Food Biological Safety Control, Food Safety and Technology Research Center, Hong Kong PolyU Shen Zhen Research Institute, Shenzhen, China; 2 State Key Lab of Chirosciences, Department of Applied Biology and Chemical Technology, The Hong Kong Polytechnic University, Hung Hom, Kowloon Hong Kong; 3 Second Affiliated Hospital of Zhejiang University, Hangzhou, China

## Abstract

The recent convergence of genetic elements encoding phenotypic carbapenem-resistance and hypervirulence within a single *Klebsiella pneumoniae* host strain represents a major public concern. To obtain a better understanding of the genetic characteristic of this emerging ‘superbug’, the complete genomes of 3 isolates of ST11 carbapenemase-producing hypervirulent *K. pneumoniae* were generated using the Oxford nanopore MinION platform. Comparative whole-genome analysis identified 13 SNPs and 3 major regions of indels in the chromosomes of the clonally disseminated isolates. IS*Kpn18*-mediated disruption in the *mgrB* gene, which was associated with colistin resistance, was identified in two later strains, leading to the emergence of hypervirulent *K. pneumoniae* that was simultaneously colistin- and carbapenem-resistant. Five plasmids were recovered from each isolate, including a 178 Kb IncHI1B/FIB-type *rmpA2*-bearing virulence plasmid, a 177.5 Kb IncFII/R self-transferable *bla*_KPC-2_-bearing MDR plasmid, a 99.7 Kb Incl1 plasmid and two ColRNAI-type plasmids of sizes of 11.9 and 5.6 Kb, respectively. The presence of homologous regions between the non-conjugative virulence plasmid and conjugative *bla*_KPC-2_-bearing MDR plasmid suggests that transmission of the virulence plasmid from ST23 *K. pneumoniae* to ST11 CRKP may be mediated by the co-integrated transfer of these two plasmids. Emergence of colistin-resistant and carbapenemase-producing hypervirulent *K. pneumoniae* strains further emphasizes the urgency for the establishment of a coordinated global program to eradicate hypervirulent and/or pan-drug-resistant strains of *K. pneumoniae* from clinical settings and the community.

## Introduction

Hypervirulent *Klebsiella pneumoniae* (hvKP), primarily associated with sequence type (ST) 23, and carbapenem-resistant (CR) *K. pneumoniae*, mostly belonging to ST11, represent two major types of clinically significant pathogens in China^1,2^. Recently, a genetically and phenotypically convergent clone that simultaneously exhibits hypervirulence and carbapenem resistance, namely CR-hvKP, has emerged. Apart from the ST 23 and ST11 strains, CR-hvKP strains of less common genetic types such as ST65, ST1797, ST43, and ST231, have also been identified^3–8^. These strains are considered real ‘superbugs’ as they are not only hypervirulent and multidrug resistant, but also highly transmissible, causing severe and often fatal infections in both hospital settings and the community^6^. According to the announced draft genomes of representative CR-hvKP isolates, such convergent clones form as a result of either horizontal transfer of resistance plasmids to hypervirulent strains or through acquisition of the pLVPK-like virulence plasmid by carbapenemase-producing strains^3,6^. In a previous study, we reported a fatal outbreak of ST11 CR-hvKP strains in a Chinese hospital and revealed the genomic characteristics of five representative causative strains based on Illumina short read sequences^6^. The strains were demonstrated to belong to one single clone with slightly different PFGE patterns (one or two band differences) among the clone strains, suggesting the possibility that genomic rearrangement frequently occurs among these strains^6^. However, the complete genome sequence of the CR-hvKP strain is not currently available, preventing comprehensive and detailed genetic analysis of this ‘superbug’.

Recent advances in third generation sequencing platforms, including the single molecule real-time (SMRT) sequencing (Pacific Biosciences), nanopore sequencing (Oxford Nanopore Technologies), etc., have provided effective tools for delineating the complete sequences of bacterial genomes^9^. In particular, nanopore MinION sequencing offers the advantage of being a timesaving procedure for library preparation and reliable data analysis^10^. Through hybrid assembly with short-read sequencing data and MinION nanopore reads, high-quality and completely assembled sequences can be generated^10^. To track microevolution events among the outbreak strains as well as provide reference sequences for genome-based hvKP studies, we performed long read sequencing with the portable Oxford Nanopore MinION device and delineated the complete genetic structures of these ST11 CR-HvKP ‘superbugs’.

## Results

Between late February and April 2016, five CR *K. pneumoniae* strains that caused fatal infections in five patients were identified at the integrated ICU of the Second Affiliated Hospital of Zhejiang University (Hangzhou, China)^6^. Phenotypic analysis showed that the isolates were string test-positive, CR and hypervirulent. Analysis of short-read sequencing data suggested that the isolates originated from a single clone, which belonged to ST11, serotype K47 *K. pneumoniae*, and harbored the *bla*_KPC-2_ gene and a pLVPK-like virulence plasmid^6^. ST11, being the dominant clone of KPC-producing *K. pneumoniae* in China, comprises at least three clusters distinguishable mainly by the serotypes^2,11^. Through acquiring the pLVPK-like virulence plasmid, the ST11 CR-hvKP clone emerged as a real ‘superbug’, that is simultaneously hypervirulent, multidrug resistant, and highly transmissible^3,6^. Retrospective studies have demonstrated that CR-hvKP actually emerged sometime before 2015 and has since become detectable in different regions of Asia, including Mainland China, Hong Kong and India, indicating that hvKP may undergo worldwide dissemination in the near future^5,12,13^.

### General characteristics of the ST11 CR-hvKP genomes

PFGE analysis in a previous study revealed that *K. pneumoniae* isolates 2, 3, and 5 are more closely related and exhibit almost identical PFGE patterns when compared with isolates 1 and 4^6^, thus only one of the three isolates (*K. pneumoniae* 5) as well as isolates 1 and 4 were subjected to long-read sequencing. The complete genome of each of the three isolates was found to contain a chromosome of 5.4 Mbp in size, and 5 plasmids ranging from 5 to 178 Kbp. The overall G + C content of the three chromosomes was 57.4%, with ~5200 coding sequences (CDSs) in each isolate (Supplementary table S1). The three CR-hvKP strains, despite being members of the same clone, were found to harbor different numbers of insertion sequences (ISs) and prophages. On the other hand, the antimicrobial resistance (AMR) genes *aadA2*, *sul1*, *bla*_SHV-11_ were detectable in all three of the chromosomes, of which *bla*_SHV_ was a core chromosomal gene in *K. pneumoniae*, and *sul1* and *aadA2* were integrated into the chromosome via insertion sequence (IS*26*)-mediated transposition. The chromosomes of the test strains were also found to harbor the previously described yersinabactin system, the type 1 fimbriae cluster *fimABCDE*, and the type 3 fimbriae cluster *mrkABCD*, which encode a moderate level of virulence in *K. pneumoniae*.

### Comparison of the ST11 CR-hvKP chromosomal sequences

**Fig. 1:**
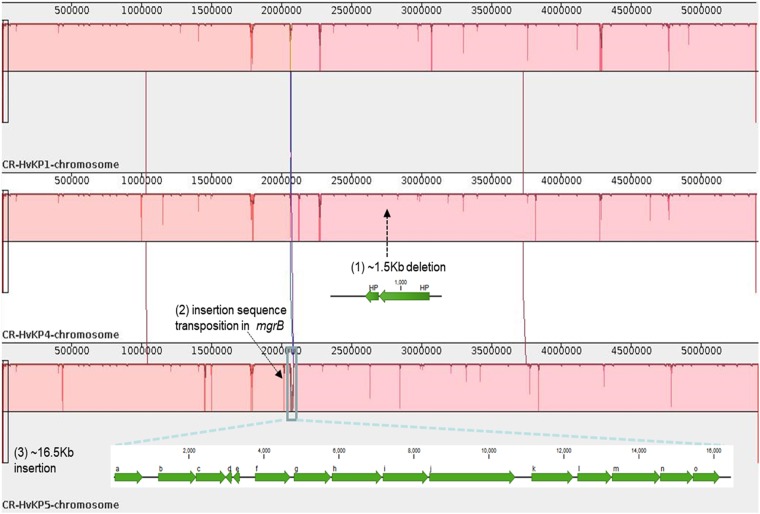
Comparison of the ST11 CR-hvKP genomes. Major regions of divergence are labeled, including: (1) An 1.5-Kb deletion in the *K. pneumoniae* 4 genome with two genes encoding hypothetical proteins (indicated with a black arrow); (2) disruption of the *mgrB* gene by insertion sequence (IS*Kpn18*) in the *K. pneumoniae* 5 genome (indicated with a black arrow); (3) A 16.5-Kb region of the *K. pneumoniae* 5 genome (indicated by a blue rectangle) is absent in the other two ST11 *K. pneumoniae* genomes. Predicted genes within this region are indicated: (a) peptidase; (b) fatty acid desaturase; (c) phosphate ABC transporter substrate-binding protein; (d) nitrilotriacetate monooxygenase; (e) hypothetical protein; (f) transcriptional regulator TdcA; (g) bifunctional threonine ammonia-lyase/L-serine ammonia-lyase TdcB; (h) threonine/serine transporter TdcC; (i) propionate kinase; (j) formate C-acetyltransferase; (k) branched-chain amino acid ABC transporter substrate-binding protein; (l) branched-chain amino acid ABC transporter permease, LivH; (m) branched-chain amino acid ABC transporter permease, LivM; (n) ABC transporter ATP-binding protein, LivG; (o) ABC transporter ATP-binding protein, LivF

**Fig. 2:**
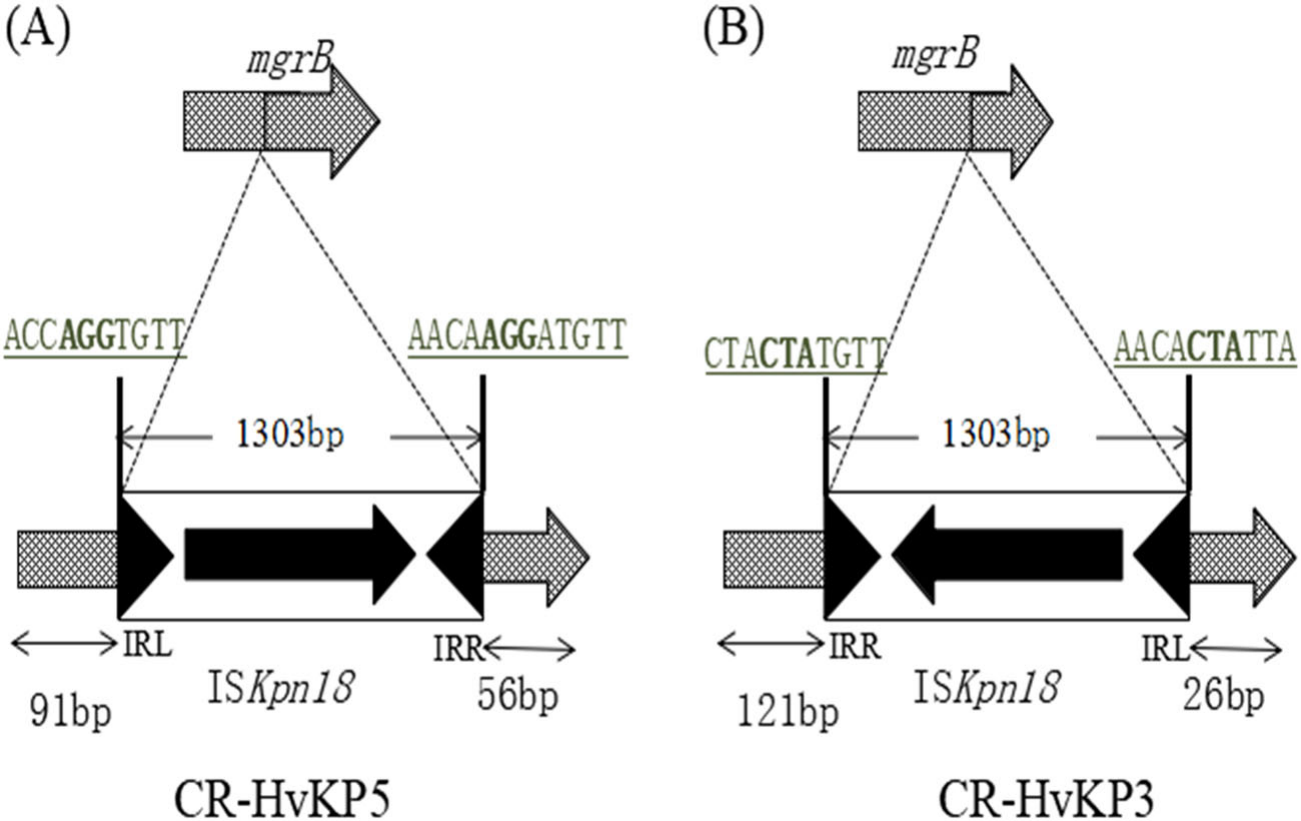
Schematic representation of the two insertion events that occurred in the *mgrB* gene. The *mgrB* gene was truncated by IS*Kpn18* in both *K. pneumoniae* 5 (**a**) and *K. pneumoniae* 3 (**b**) strains with opposite directions at different positions. Target site duplications are underlined with bold letters. The left and right inverted repeats (IRL and IRR) of IS*Kpn18* are represented as black triangles

To investigate the population structure of the ST11 isolates in detail, we studied the chromosomal SNPs among the three fully sequenced isolates. By aligning the raw Illumina sequencing reads of isolates 1, 4, and 5 against the complete sequence of *K. pneumoniae* isolate 1, a total of 13 synonymous and nonsynonymous SNPs were identified (Table 1). In addition to SNPs, the ST11 CR-hvKP chromosomes mainly differ from each other in three regions of deletion and insertions (Fig. 1), one of which is a 16.5-Kb genomic region that contains 15 predicted ORFs at position 2.06 Mb in the *K. pneumoniae* 5 genome. A BLASTN search revealed that this element shares 100% identity with several fragments in various other *K. pneumoniae* genomes with 100% coverage, including the genomes of the ST11 strains SWU01 and GD4^11,14^, suggesting that this element is widely distributed, but is not necessarily a core cassette in *K. pneumoniae* genomes. Genes related to threonine and serine metabolism and transport during anaerobic growth (the *tdc* operon) and a cluster encoding branched-chain amino acid ABC transporter (the *liv* operon) were annotated in this 16.5-Kb element^15,16^. Additionally, a 1.5-Kb region found at position 2.7 Mb of *K. pneumoniae* 1 and 5 was absent in the genome of *K. pneumoniae* 4. This element was predicted to encode two hypothetical proteins. Furthermore, truncation of the *mgrB* gene by IS*Kpn18* at position 2.0 Mb was detected in the genome of *K. pneumoniae* 5. To verify the effect of *mgrB* gene truncation, colistin susceptibilities of the five isolates were tested. Strains *K. pneumoniae* 3 and 5 were both colistin-resistant, with MICs of 64 µg/ml. Genetic analysis revealed that the *mgrB* genes in the two strains were both truncated by IS*Kpn18*, but the positions (nt91 and nt121) and orientations of the two insertions were different (Fig. 2). To track the origin of this IS element, which frequently causes colistin resistance in *K. pneumoniae*, we searched the chromosome of these three strains for the presence of this IS element. Our results indicated that the IS*Kpn18* element was located at position 4.4 Mb of the *K. pneumoniae* chromosome in these three strains.

**Table 1:** List of chromosomal SNP differences among the clonal CR-hvKP outbreak strains tested

Position in reference	CR-HvKP1 (Reference)	CR-HvKP4	CR-HvKP5	Product	Effect with DNA (c.) and protein (p.) changes
53198	A	G	G	–	
547060	A	C	C	Lipoprotein nlpI precursor	Missense variant; c.115 A > C p.Lys39Gln
621416	A	A	T	Phosphoenolpyruvate-dihydroxyacetone phosphotransferase (EC 2.7.1.121), subunit DhaM	Missense_variant; c.992 T > A p.Val331Asp
769687	A	C	C	Antirestriction protein	Stop_lost & splice_region_variant; c.740 A > C p.Ter247Serext*?
1781309	A	C	C	–	
1783244	A	C	C	–	
2179080	T	T	G	–	
4604288	G	T	G	Hypothetical protein	Missense variant; c.76 C > A p. Gln26Lys
4766271	A	G	A	–	
4766275	G	A	G	–	
4766278	G	A	G	–	
4767184	G	A	G	–	
4767185	A	G	A	–	

Nucleotide and amino acid changes in DNA and protein sequences of *K. pneumoniae* strains CR-HvKP4 and CR-HvKP5, with reference to the *K. pneumoniae* CR-HvKP1 chromosome sequence; The symbol ‘–’ in the column “product” represents non-coding regions in the sequence

### Extrachromosomal elements of the ST11 CR-hvKP isolates

**Fig. 3:**
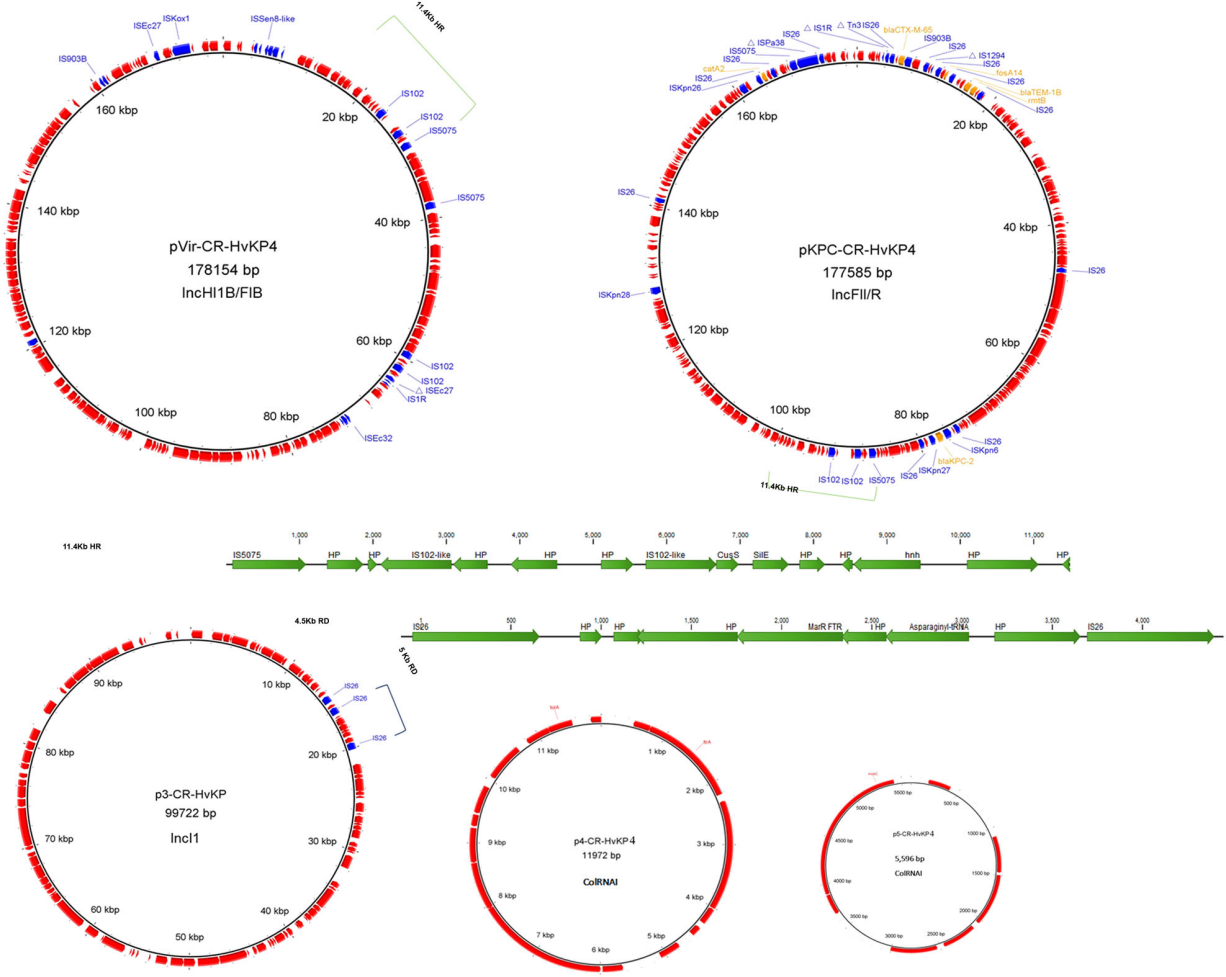
Plasmids from the ST11 CR-hvKP isolates. Each of the three ST11 isolates carried five plasmids, with sizes of 178, 177.5, 99.7, 11.9, and 5.6 Kb. Insertion sequences and antimicrobial resistance genes are annotated with blue and yellow fonts, respectively. Plasmid name, size, and replicon types are depicted in the middle of each circle. The 11.4-Kb homologous region (HR) in the virulence plasmid and MDR plasmid, and 4.5-Kb region of divergence (RD) in the 99.7-Kb plasmid are highlighted

Each of the three ST11 CR-hvKP isolates was found to harbor five plasmids, the sizes of which were 178, 177.5, 99.7, 11.9, and 5.6 Kb, respectively (Fig. 3, Supplementary table S2). Due to the high degree of sequence similarity of the plasmid contents in the three hvKP isolates, plasmids in strain *K. pneumoniae* 4 were chosen as representative sequences in subsequent analyses. The pLVPK-like virulence plasmid (pVir-CR-HvKP, 178 Kb) was only slightly larger than another *bla*_KPC-2_-bearing MDR plasmid in the same strain (pKPC-CR-HvKP, 177.5 Kb). A BLASTN search in the NCBI database revealed that pVir-CR-HvKP4 exhibited 99% identity to the 219 Kb typical virulence plasmid in *K. pneumoniae*, pLVPK (AY378100), with 90% coverage. As reported previously, a 58.8-Kb element that encodes hypothetical proteins located between two genes in position 75.6 Kb of pLVPK was absent in pVir-CR-HvKP4 (Supplementary Figure S1)^6^. This element carries 71 predicted ORFs, including *rmpA*, *iroBCDN* (salmochelin), *fecIRA*-like ferric citrate uptake-related genes, and 40 other genes with unknown functions. Other major differences between the two plasmids include the 11.8-Kb and 6.7-Kb fragments carried by pVir-CR-HvKP4 but not by pLVPK. This 11.8-Kb fragment, located between the *cus* locus and a hypothetical gene, shares 100% identity and 100% coverage with a fragment in the 231 Kb *K. pneumoniae* virulence plasmid, pSGH10^17^. It carries 20 ORFs, including multiple insertion sequences (an IS*1R*, a truncated IS*Ec27* and 2 IS*102*-like sequences) and genes encoding hypothetical proteins. Likewise, the 6.7-Kb element carries an IS*5075*, two IS*102*-like insertion sequences and genes of unknown functions. The 6.7-Kb element, along with a downstream 4.7-Kb fragment of pSGH10, exhibited 99% identity and 99% coverage with an 11.4-Kb fragment in plasmid pKPC-CR-HvKP4 (Fig. 3). Additionally, inversion at a 27.6-Kb fragment carrying multiple heavy metal resistance genes (the *sil*, *pco* and *pbr* gene clusters) was detected in plasmid pVir-CR-HvKP4 when compared with pLVPK. The *pbr* locus was located in a Tn*3*-like transposon, which could potentially contribute to the genetic rearrangement of these virulence plasmids.

The 177.5-Kb *bla*_KPC-2_-bearing plasmid was found to belong to the class of IncFII/R-type self-transferable MDR plasmid and carry the resistance genes *bla*_KPC-2_, *bla*_CTX-M-65_, *bla*_TEM-1b_, *rmtB*, *catA2* and *fosA14*. This plasmid shares 94% coverage and 99% identity to the *K. pneumoniae* IncFII/R type plasmid pKPGD4 (GenBank accession: CP025952), which was previously isolated from an ST11 isolate and also found to harbor the aforementioned resistance genes^11^. The major difference between plasmids pKPC-CR-HvKP4 and pKPGD4 lies in the 11.4-Kb element, which is carried by the plasmids pKPC-CR-HvKP4 and pVir-CR-HvKP4, but absent in pKPGD4 (Fig. 3, supplementary Figure S2).

The 99.7-Kb Incl1-type, non-MDR plasmid p3-CR-HvKP4 exhibits 94% coverage and 99% identity to the 115.7-Kb *E. coli* Incl1-type plasmid pS68 (KU130396). Plasmid p3-CR-HvKP4 shares 94 Kb of its sequence with pS68. However, a 22.4-Kb region of pSa68 bordered by IS*26* at both ends, which carries multiple drug resistance genes (*aadA1*, *rmtE*, *ermB*), is lost in the 99.7-Kb plasmid. Instead, a 4.5-Kb region that includes 9 ORFs was integrated into the pS68 backbone. This region encodes a MarR family transcriptional regulator, an asparaginyl-tRNA synthetase, two IS*26* transposases and 5 hypothetical proteins. It is 100% identical to the 277-Kb plasmid pRpNDM1-1 (JX515588), which was previously isolated from a *Raoultella planticola* isolate^18^.

Furthermore, all ST11 CR-hvKP isolates were found to carry two ColRNAI plasmids of sizes 11.9 and 5.6-Kb. The 11.9-Kb plasmid was found to exhibit 99% identity to an unnamed plasmid (CP023932) isolated from *K. pneumoniae* strain FDAARGOS_443 with 100% coverage, and harbor 13 ORFs encoding colicin E3, a mobilization protein and hypothetical proteins. The 5.6-Kb plasmid was commonly detected in *K. pneumoniae*, and encodes the RelE/StbE replicon stabilization toxin/antitoxin system and seven hypothetical proteins.

## Discussion

The *K. pneumoniae* genome is known to undergo constant evolution, with an estimated 10.1 nucleotide substitutions/genome/year^19,20^. Despite being members of a single clone that were indistinguishable by phylogenetic analysis and simple sequence alignments (Fig. 1), the outbreak-related ST11 isolates differ by 13 specific nucleotides termed SNPs. Given that the three isolates were sampled within a 2-month-period, we estimated that the ST11 clone could have evolved at a velocity higher than that reported previously^20^. Apart from SNPs, mobile genome elements including plasmids, phages, integrated conjugative elements (ICEs) and insertion sequences (ISs), which have been demonstrated to drive genomic evolution in *K. pneumoniae*, were not identical in all three isolates, indicating that active genomic rearrangement occurred during the transmission process^21^. In line with this finding is that three major indels were detected among the three isolates, including genes related to amino acid metabolism and transportation, hypothetical genes and truncation of the *mgrB* gene by IS*Kpn18*. MgrB is a negative regulator of the PhoPQ two-component regulatory system, which is associated with colistin resistance in *K. pneumoniae*^22^. Inactivation of the *mgrB* gene was demonstrated to be a common cause of colistin resistance in clinical carbapenemase-producing *K. pneumoniae* strains^23^. A previous study reported that colistin resistance in a ST258 *bla*_KPC-2_-bearing *K. pneumoniae* strain was mediated by insertion of IS*Kpn18* in the *mgrB* gene^24^. Also, insertion of IS*Kpn18* in the *ramR* gene, which is associated with tigecycline resistance in *K. pneumoniae*, has been reported^25^. A previous study has demonstrated that IS*Kpn18* is almost entirely restricted to strains of CG258 and the proportion of intragenic insertion of IS*Kpn18* is over 80% in *K. pneumoniae*^26^. All evidence indicates that IS*Kpn18* can readily cause intragenic insertion in *K. pneumoniae*. Because of this inactivation, two later strains, *K. pneumoniae* 3 and 5, have emerged as real ‘superbugs’; they are simultaneously carbapenem- and colistin- resistant, hypervirulent and transmissible and could not be treated with last-line-of-defense antibiotics.

Plasmids play pivotal roles in the dissemination of virulence determinants and antimicrobial resistance phenotypes. In this study, five plasmids with sizes of 178, 177.5, 99.7, 11.9, and 5.6 Kb were identified in each of the three sequenced genomes. The 178-Kb plasmid pVir-CR-HvKP4 is a pLVPK-like virulence plasmid. pLVPK is known to harbor genes encoding capsular polysaccharide synthesis regulators (*rmpA* and *rmpA2*) and iron-acquisition systems (*iucABCDiutA* and *iroBCDN* siderophore gene clusters), which are associated with enhanced virulence potential and genes related to heavy metal (copper, silver, lead, and tellurite) resistance^27^. Despite the absence of a 58.8-Kb element carrying multiple virulence-related genes from pVir-CR-HvKP4 compared with plasmid pLVPK, the plasmid does contribute to the virulence of the host strain. Curing of plasmid pVir-CR-HvKP4 resulted in a negative string test phenotype and substantially reduced virulence potential of the host strain^6^. Multiple mobile elements were detected on pVir-CR-HvKP4 and genetic rearrangements were found compared with other related virulence plasmids, which suggests that this virulence plasmid has undergone constant evolution by acquisition and loss of DNA fragments through horizontal gene transfer mediated by different IS elements. Future research should study whether variation in the genetic organization of virulence plasmids leads to the divergence in the virulence potential of *K. pneumoniae* isolates. The 177.5-Kb *bla*_KPC-2_-bearing conjugative plasmid pKPC-CR-HvKP4 contributed to the carbapenem resistance phenotypes of the host strains^6^. Plasmids that possess the pKPC-CR-HvKP4 backbone are highly prevalent among *bla*_KPC-2_-bearing *K. pneumoniae* strains in China according to previous studies^11^. A 11.4-Kb element on pKPC-CR-HvKP4 was homologous to that on pVir-CR-HvKP4, suggesting that it might contribute to the transmission of a virulence plasmid that is not conjugative from ST23 HvKP to ST11 *K. pneumoniae* with the help of the conjugative *bla*_KPC-2_-bearing plasmid, likely through plasmid cointegration. The details of the mechanisms of transmission of virulence plasmids require further investigation.

In summary, this study delineated genetic microevolution events that occurred among clonal ST11 hypervirulent, CR *K. pneumoniae* strains by using the nanopore MinION sequencing platform. We identified various chromosomal SNPs, indels and unique genetic features of the plasmids harbored by the tested strains. To our knowledge, this is not only the first report of the complete sequence of a ST11 CR-hvKP clone but also the first report on the emergence of colistin- and CR hypervirulent *K. pneumoniae* strains. In addition, through sequence analysis of their extrachromosomal plasmids, the mechanisms of transmission of virulence plasmids from ST23 HvKP to ST11 CRKP might be revealed. Coordinated efforts are required to eradicate such hypervirulent and pan-drug-resistant strains, which may otherwise pose a worldwide public health threat in the near future.

## Material and Methods

### Strains and susceptibility tests

Source information of the five outbreak strains studied in this work can be found in our previous study^6^. The colistin susceptibility of the isolates was determined and interpreted as previously described^28^.

### Whole genome sequencing and assembly

Three of the five outbreak-related *K. pneumoniae* isolates (1, 4, and 5) which displayed different PFGE patterns as described in our previous study were selected for whole genome sequencing using the nanopore MinION device (Oxford Nanopore Technologies, Oxford, United Kingdom), according to previously described methods^10^. Briefly, genomic DNA was extracted from overnight cultures using the PureLink Genomic DNA Mini Kit (Invitrogen, Carlsbad, CA, USA). MinION libraries of the test isolates were prepared using the SQK-RBK001 nanopore sequencing kit (version R9.4) according to the manufacturer’s instructions. Illumina sequencing reads for the isolates were derived from the previous study^6^. The hybrid read set (both Illumina and nanopore reads) for the genomes of each isolate was assembled using Unicycler (v0.4.0) with manual curation as necessary^29^. The complete genome sequences were annotated with the RAST tool^30^ and Prokka^31^. The chromosome was adjusted, with *dnaA* being the first gene.

### Bioinformatics analysis

Sequences and annotations were visualized and edited with the CLC Genomics Workbench (version 9.0). ICEs, acquired antibiotic resistance genes, plasmid replicons, ISs, virulence-related genes and phage-associated regions were identified using previously described methods^11^. Single-nucleotide polymorphisms (SNPs) were detected using Snippy v3.2 (https://github.com/tseemann/snippy) by mapping the Illumina sequence reads of the three CR-hvKP isolates to the complete chromosome sequence of isolate *K. pneumoniae* 1. A minimum coverage depth of 10 and base call stringency of 90% were selected for SNP detection. The identified SNPs were manually inspected and edited. ProgressiveMauve (version 2.4.0) was applied to compare the chromosomal architectures^32^. BLASTN was conducted to screen for sequences homologous to the sequenced plasmids in the NCBI database. Comparison between homologous plasmids was conducted using EasyFig 2.1^33^. Plasmid maps were generated using the BLAST Ring Image Generator **(**BRIG) v0.95^34^.

### Data availability

All sequencing data have been deposited in GenBank under the accession numbers SAMN09487516, SAMN09487517, and SAMN09487518.

## Electronic supplementary material

Supplementary materials

## References

[1] Struve C (2015). Mapping the Evolution of Hypervirulent *Klebsiella pneumoniae*. mBio.

[2] Qi Y (2011). ST11, the dominant clone of KPC-producing *Klebsiella pneumoniae* in China. J. Antimicrob. Chemother..

[3] Chen, L. & Kreiswirth B. N. Convergence of carbapenem-resistance and hypervirulence in *Klebsiella pneumoniae*. *Lancet Infect. Dis*., **18**, 2–3 (2018). 10.1016/S1473-3099(17)30517-0 28864028

[4] Zhang R (2015). Emergence of carbapenem-resistant serotype K1 hypervirulent *Klebsiella pneumoniae* strains in China. Antimicrob. Agents Chemother..

[5] Shankar, C. et al. Draft genome sequences of three hypervirulent carbapenem-resistant *Klebsiella pneumoniae* isolates from bacteremia. *Genome Announc.*, **4**, e01081–16 (2016). 10.1128/genomeA.01081-16 PMC5146430 27932638

[6] Gu, D. et al. A fatal outbreak of ST11 carbapenem-resistant hypervirulent *Klebsiella pneumoniae* in a Chinese hospital: a molecular epidemiological study. *Lancet Infect. Dis.*, 2017. 10.1016/S1473-3099(17)30489-9 28864030

[7] Yao B (2015). Clinical and molecular characteristics of multi-clone carbapenem-resistant hypervirulent (hypermucoviscous) *Klebsiella pneumoniae* isolates in a tertiary hospital in Beijing, China. Int. J. Infect. Dis..

[8] Zhang Y (2015). Emergence of a hypervirulent carbapenem-resistant *Klebsiella pneumoniae* isolate from clinical infections in China. J. Infect..

[9] Goodwin S, McPherson JD, McCombie WR (2016). Coming of age: ten years of next-generation sequencing technologies. Nat. Rev. Genet..

[10] Li R (2018). Efficient generation of complete sequences of MDR-encoding plasmids by rapid assembly of MinION barcoding sequencing data. Gigascience.

[11] Dong, N. et al. Genome analysis of Clinical Multilocus Sequence Type 11 *Klebsiella pneumoniae* from China. *Microb. Genom*., 10.1099/mgen.0.000149 (2018). 10.1099/mgen.0.000149 PMC5857376 29424684

[12] Wong MHY (2018). Emergence of carbapenem-resistant hypervirulent *Klebsiella pneumoniae*. Lancet Infect. Dis..

[13] Du P, Zhang Y, Chen C (2018). Emergence of carbapenem-resistant hypervirulent *Klebsiella pneumoniae*. Lancet Infect. Dis..

[14] Zhang L (2017). Whole-genome sequence of a carbapenem-resistant hypermucoviscous *Klebsiella pneumoniae* isolate SWU01 with capsular serotype K47 belonging to ST11 from a patient in China. J. Glob. Antimicrob. Resist..

[15] Lai YC, Peng HL, Chang HY (2001). Identification of genes induced in vivo during *Klebsiella pneumoniae* CG43 Infection. Infect. Immun..

[16] Bronner D, Clarke BR, Whitfield C (1994). Identification of an ATP‐binding cassette transport system required for translocation of lipopolysaccharide O‐antigen side‐chains across the cytoplasmic membrane of *Klebsiella pneumoniae* serotype O1. Mol. Microbiol..

[17] Lam, M. M. et al. Population genomics of hypervirulent *Klebsiella pneumoniae* clonal group 23 reveals early emergence and rapid global dissemination. *bioRxiv*, 10.1101/225359 (2017). 10.1038/s41467-018-05114-7 PMC6045662 30006589

[18] Li J (2014). Sequential isolation in a patient of Raoultella planticola and Escherichia coli bearing a novel ISCR1 element carrying blaNDM-1. PLoS One.

[19] Mathers AJ (2015). *Klebsiella pneumoniae* carbapenemase (KPC)-producing K. pneumoniae at a single institution: insights into endemicity from whole-genome sequencing. Antimicrob. Agents Chemother..

[20] Zautner AE (2017). Monitoring microevolution of OXA-48-producing *Klebsiella pneumoniae* ST147 in a hospital setting by SMRT sequencing. J. Antimicrob. Chemother..

[21] Chen L (2014). Carbapenemase-producing *Klebsiella pneumoniae*: molecular and genetic decoding. Trends Microbiol..

[22] Poirel L (2014). The mgrB gene as a key target for acquired resistance to colistin in *Klebsiella pneumoniae*. J. Antimicrob. Chemother..

[23] Cannatelli A (2014). MgrB inactivation is a common mechanism of colistin resistance in KPC carbapenemase-producing *Klebsiella pneumoniae* of clinical origin. Antimicrob. Agents Chemother..

[24] Arena F (2016). Colistin resistance caused by inactivation of the MgrB regulator is not associated with decreased virulence of sequence type 258 KPC carbapenemase-producing *Klebsiella pneumoniae*. Antimicrob. Agents Chemother..

[25] Hudson CM (2014). Resistance determinants and mobile genetic elements of an NDM-1-encoding *Klebsiella pneumoniae* strain. PLoS One.

[26] Adams MD, Wright MS, Bishop B (2016). Quantitative assessment of insertion sequence impact on bacterial genome architecture. Microbial Genomics.

[27] Chen YT (2004). Sequencing and analysis of the large virulence plasmid pLVPK of *Klebsiella pneumoniae* CG43. Gene.

[28] Li R (2017). Complete genetic analysis of plasmids carrying mcr-1 and other resistance genes in an *Escherichia coli* isolate of animal origin. J. Antimicrob. Chemother..

[29] Wick RR (2017). Unicycler: resolving bacterial genome assemblies from short and long sequencing reads. PLoS. Comput. Biol..

[30] Overbeek R (2014). The SEED and the Rapid Annotation of microbial genomes using Subsystems Technology (RAST). Nucleic Acids Res..

[31] Seemann T (2014). Prokka: rapid prokaryotic genome annotation. Bioinformatics.

[32] Darling AC (2004). Mauve: multiple alignment of conserved genomic sequence with rearrangements. Genome Res..

[33] Sullivan MJ, Petty NK, Beatson SA (2011). Easyfig: a genome comparison visualizer. Bioinformatics.

[34] Nabil-Fareed Alikhan NKP, Nouri LBenZakour, Beatson ScottA (2011). BLAST Ring Image Generator (BRIG): simple prokaryote genome comparisons. BMC Genomics.

