# Supplementary material for: Tracking microevolution events among ST11 carbapenemase-producing hypervirulent Klebsiella pneumoniae outbreak strains
Source: Emerg Microbes Infect. 2018 Aug 12;7:146. doi: 10.1038/s41426-018-0146-6 (PMC6087712; doi:10.1038/s41426-018-0146-6)
Supplement: Supplementary file 1 — Supplementary materials [file 41426_2018_146_MOESM1_ESM.pdf]

Supplementary materials

Tracking microevolution events among ST11 carbapenemase-producing hypervirulent *Klebsiella pneumoniae* outbreak strains

Ning Dong [1,2], Xuemei Yang[1,2], Rong Zhang [3], Edward Wai-Chi Chan[2], Sheng Chen [1,2,*]

[1]Shenzhen Key lab for Food Biological Safety Control, Food Safety and Technology Research Center, Hong Kong PolyU Shen Zhen Research Institute, Shenzhen, P. R. China;

[2]State Key Lab of Chirosciences, Department of Applied Biology and Chemical Technology, The Hong Kong Polytechnic University, Hung Hom, Kowloon, Hong Kong;

[3]Second Affiliated Hospital of Zhejiang University, Hangzhou, China;

Corresponding authors: Sheng Chen, sheng.chen@polyu.edu.hk

Keywords: *Klebsiella pneumoniae*, ST11, carbapenem resistance, hypervirulence, evolution, genome sequence, Nanopore MinION sequencing

Supplementary table 1. Genome statistics of the ST11 CR-hvKP *K. pneumoniae* strains.

	K. pneumoniae 5	*K. pneumoniae* 4	*K. pneumoniae* 1
Chromosome Size (bp)	5,408,302	5,388,721	5,390,059
G+C content (%)	57.4	57.4	57.4
Number of CDS	5207	5205	5197
number of rRNA	25	24	24
number of tRNA	76	82	73
Prophages	9	9	8
number of ICE	2	2	2
number of IS	44	44	43
IS*Plu15*	6	6	6
IS*407*	2	2	2
IS*903*	1	1	1
IS*5*	14	15	12
IS*3*	2	1	1
IS*6*	2	2	3
IS*1*	3	3	3
IS*481*	4	4	3
IS*150*	5	5	5
IS*1182*	1	1	1
IS*1380*	2	2	4
IS*1111*	2	2	2
chromosome-borne antimicrobial resistance genes	*aadA2*, *sul1*, $bla_{\text{SHV-11}}$	*aadA2*, *sul1*, $bla_{\text{SHV-11}}$	*aadA2*, *sul1*, $bla_{\text{SHV-11}}$
number of plasmids	5	5	5

ICE, integrative and conjugative elements; IS, insertion sequence.

Supplementary table 2. Information of plasmids in the ST11 CR-hvKP isolates.

	Length (bp)			G+C (%)			Number of CDS identified with RAST		
	CR-HvKP5	CR-HvKP4	CR-HvKP1	CR-HvKP5	CR-HvKP4	CR-HvKP1	CR-HvKP5	CR-HvKP4	CR-HvKP1
pVir-CR-HvKP*	177,694	178,154	177,790	50.5	50.5	50.5	180	179	180
pKPC-CR-HvKP*	177,587	177,585	177,591	53.1	53.1	53.1	219	218	216
p3-CR-HvKP*	99,717	99,722	99,717	49.8	49.8	49.8	104	105	107
p4-CR-HvKP*	11,970	11,970	11,970	55.6	55.6	55.6	13	13	13
p5-CR-HvKP*	5,596	5,596	5,596	51.1	51.1	51.1	9	9	9

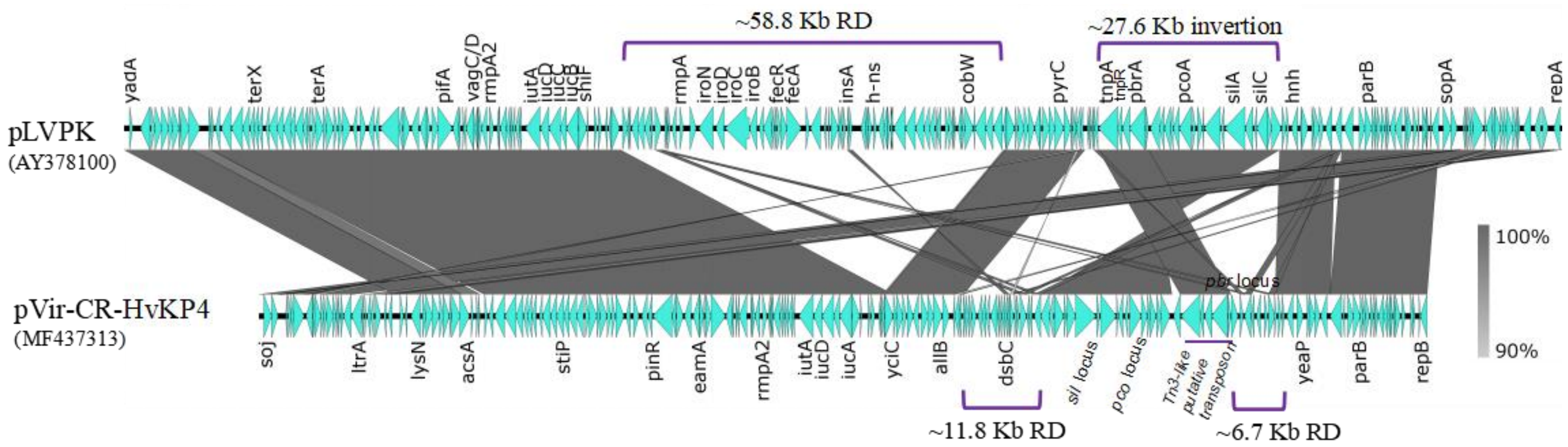

Supplementary figure S1. Comparison between virulence plasmids pVir-CR-HvKP4 (GenBank accession: MF437313) and pLVPK (GenBank accession: AY378100) in *K. pneumoniae*. Green arrows indicate the predicted genes in the plasmids. Regions of divergences (RD) were labeled with blue square brackets.

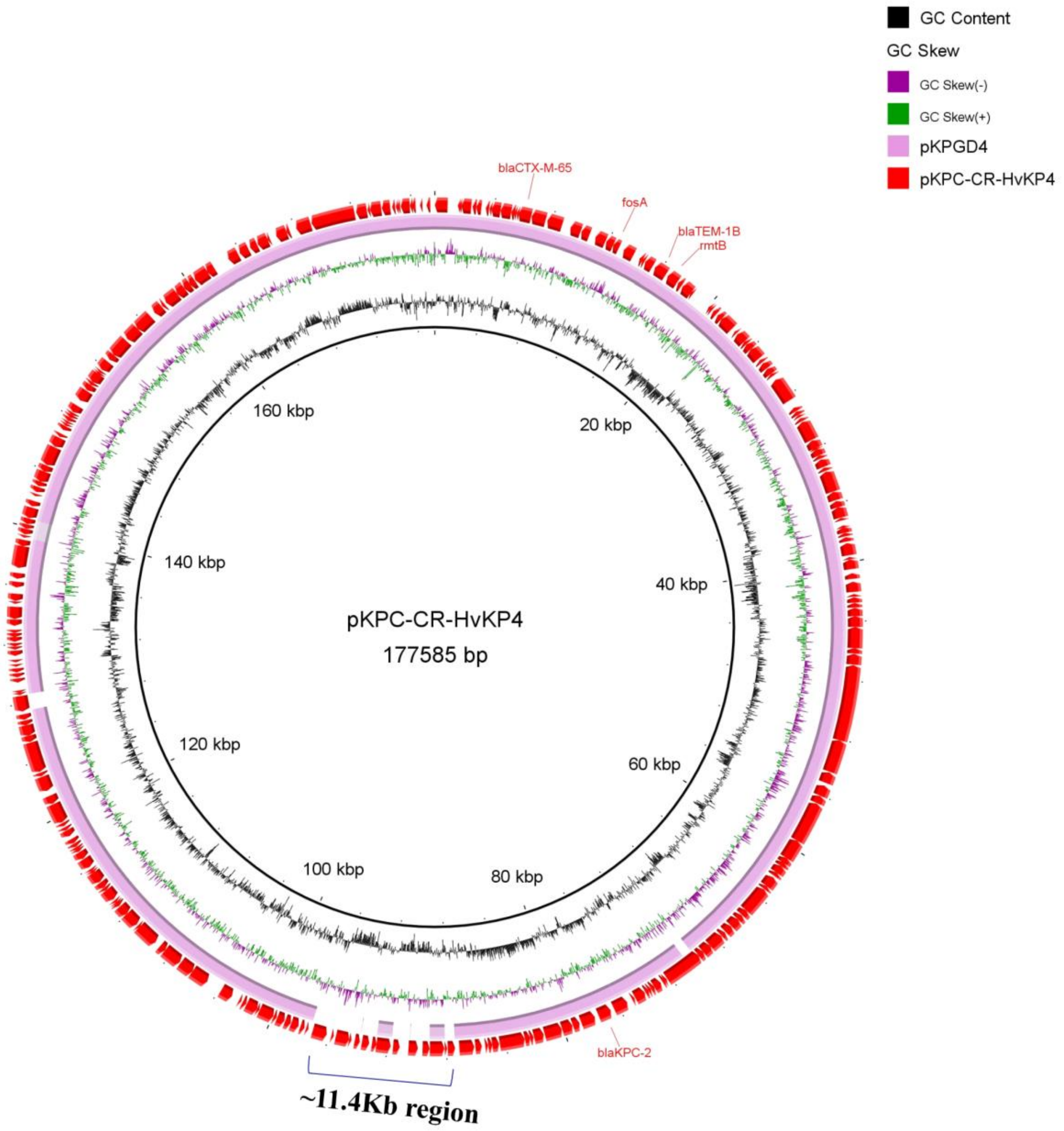

Supplementary Figure S2. Alignment of *bla*$_{KPC-2}$ bearing plasmids pKPC-CR-HvKP4 (GenBank accession: MF437312) and pKPGD4 (CP025952) in *K. pneumoniae*. The plasmid sequence of pKPC-CR-HvKP4 was used as the reference (outermost circle). Antimicrobial resistance genes and the 11.4 Kb region of divergence were labelled in the figure.
